# Initial Experience with the BioSig PURE EP™ Signal Recording System: An Animal Laboratory Experience

**DOI:** 10.19102/icrm.2017.080407

**Published:** 2017-04-15

**Authors:** Deepak Padmanabhan, Thomas Foxall, Budimir Drakulic, Chance Witt, Ammar Killu, Niyada Naksuk, Alan Sugrue, K.L. Venkatachalam, Samuel Asirvatham

**Affiliations:** ^1^Department of Cardiovascular Diseases, Mayo Clinic, Rochester, MN; ^2^BioSig Technologies, Inc., Minneapolis, MN; ^3^Department of Electrophysiology, Brigham and Womens’ Hospital, Boston, MA; ^4^Department of Cardiovascular Diseases, Mayo Clinic, Jacksonville, FL; ^5^Department of Pediatric and Adolescent Medicine, Mayo Clinic, Rochester, MN

**Keywords:** Current of injury, dynamic range, PURE EP™ system, signal processing, unipolar signal

## Abstract

Current signal recording and processing systems have come a long way since their initial inception and use. There is, however, still ample scope for improvement, not only in the troubleshooting of their limitations, but also in the expansion of the boundaries in the recording of intracardiac signals. Here, we recount our experience with the use of the PURE EP™ signal recording system (BioSig Technologies, Inc., Minneapolis, MN, USA) in the animal laboratory.

## Introduction

Electroanatomical mapping systems (EAMs) made their way into clinical practice in 1997.^1–3^ Since then, there has been a sustained advancement in the acquisition of electrograms (EGMs), mapping of cardiac chambers, the creation of ablation lesion sets, the localization of sources of abnormal cardiac activity, and an overall decrease in fluoroscopy time for ablation procedures.^4–8^ The drive to improve existing systems is an ongoing one, and has demonstrated change as our understanding of disease pathophysiology has improved. Developments in processing systems have strengthened the real-time display of information and allowed for the making of decisions on the go.^9,10^ Despite the technological advances, there have been notable limitations in the performance of current recording and mapping systems.^11,12^ Maximum procedural success has been achieved in the localization of the sources of focal arrhythmias, and in delineating re-entry circuits.^13–15^ Uncertainty in the “reality” of the signals obtained during various maneuvers, however, is still a challenge, and it is in this direction that most of the current efforts are being directed.^16^ In this paper, we describe the results of a series of experiments involving cardiac mapping and ablation in a canine model, comparing conventional electrophysiology (EP) signal processing equipment with an advanced signal processing platform called PURE EP™ (BioSig Technologies, Inc., Minneapolis, MN, USA).

### Pitfalls of current recording and mapping systems and possible solutions

**Figure 1:**
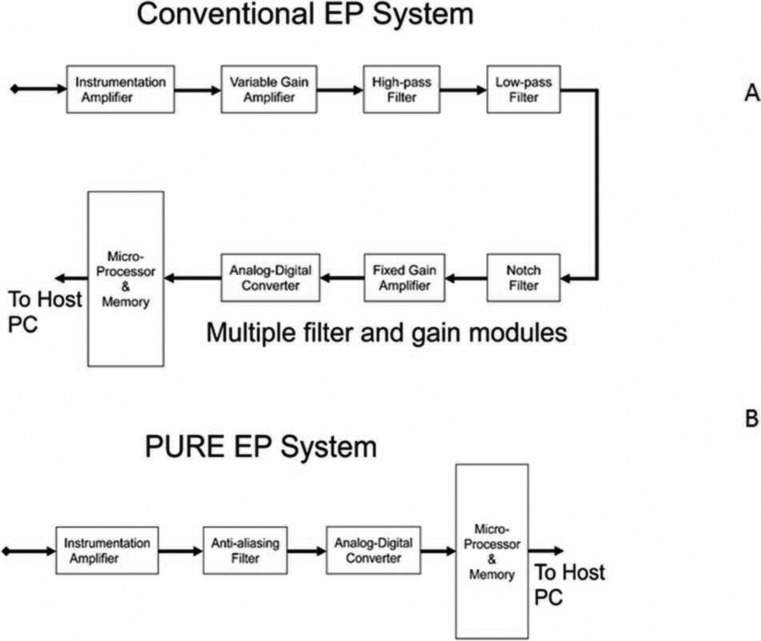
Schematic drawing to illustrate the differences in signal acquisition hardware between (A) a conventional recording system and (B) the PURE EP™ system (BioSig Technologies, Inc., Minneapolis, MN, USA).

The cardiac signal data-acquisition module of typical present-day EP recording and mapping systems is depicted in **Figure 1A**. In this architecture, various gain and filter settings are implemented in hardware. As a result, signals are recorded from a specific hardware configuration, and the original raw information generally cannot be recovered.

The PURE EP™ (BioSig Technologies, Inc., Minneapolis, MN, USA) system has the ability to allow for the recording of raw (i.e. unaltered) cardiac signals with multiple display options. This is achieved using a proprietary amplifier topology, which combines a very low-noise instrumentation amplifier (with minimal filtering to band-limit the signal) and a high-resolution 24-bit A/D converter **(Figure 1B)**. In addition, there are means to provide defibrillator protection and radiofrequency (RF) noise suppression. In this architecture, there is no need for gain switching, and the full range of input signals (± 250 mV) is digitized with optimal resolution. Raw signals acquired by the acquisition module are filtered and processed in the accompanying software, using the digital processing module. In the real-time window, waveforms of interest can be displayed as raw signals or as any combination of raw and filtered signals to enable better visualization of signals in the presence of noise and artifacts. All displayed signals are time synchronized. On the review screen, the operator has the option of opening multiple review windows, with the ability to display the results of various signal-processing algorithms, independent of the real-time tracings.

During all the experiments, we used standard electrophysiology catheters like the Blazer™ II (4-mm tip) or Blazer™ II XP (8-mm-tip) RF catheters (Boston Scientific, Marlborough, MA, USA), with 2.5-mm interelectrode spacing; or a ThermoCool^®^ SmartTouch^®^ catheter (Biosense Webster, Diamond Bar, CA, USA), with a 3.5-mm tip and an interelectrode spacing of 1-6-2 mm.

**Saturation artifact**. High gain settings in recording and mapping systems are designed to improve the visualization of very low-amplitude intracardiac signals. When presented with signals that have large amplitudes, the high gain settings may result in output signals larger than the supply voltage of the front-end amplifiers, causing saturation of the amplifiers. This shows up on the display as an artifact in the form of truncation of the signals. In addition, amplifier saturation is often followed by long recovery times before resumption of proper function, causing a further loss of signal recording during the intermediate aftermath of the saturation for a finite period. The only current solution to this is the reduction of system gain, which negatively affects the successful visualization of small signals.^17^

**Figure 2:**
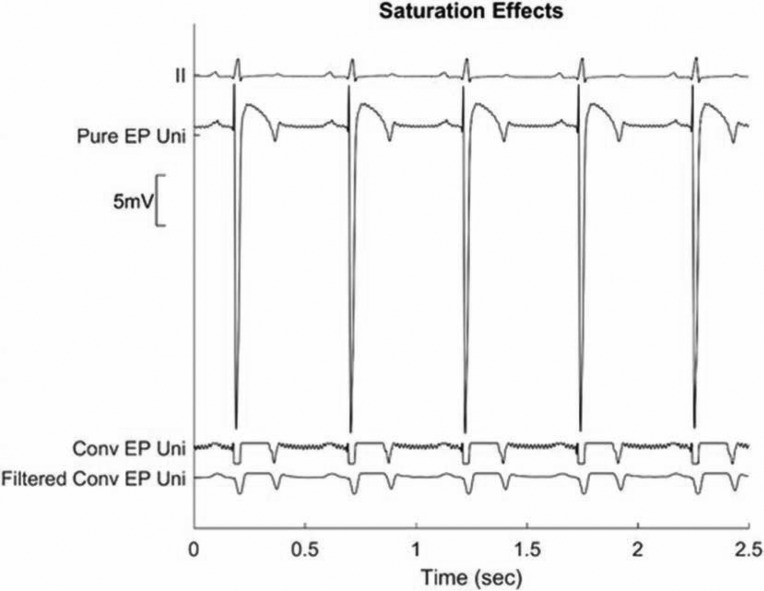
An illustration of potential problems that may occur because of saturation. Pure EP Uni: unipolar signal as recorded by the PURE EP™ system (BioSig Technologies, Inc., Minneapolis, MN, USA); Conv EP Uni: unipolar signal recorded by a conventional recorder, which has saturated; Filtered Conv EP Uni: a filtered saturated signal, which can look different than the actual morphology of the local electrogram.

In addition, the problem is magnified several-fold when pacing is attempted. Pacing signals are orders of magnitude larger than the EGMs, and are typically applied directly to the sensing electrodes, resulting in profound saturation. This, in turn, increases amplifier recovery time, compromising the ability to view or record signals for several seconds after the pacing artifact. Additionally, the pacing artifact may also be present on adjacent electrodes because of cross-talk, masking EGMs on those electrodes also. For maneuvers like entrainment and differential output pacing, this causes inaccuracies, since there is always obscuration of the local EGMs by the pacing artifact. **Figure 2** shows an example of saturation adversely affecting displayed signals. The lead labeled “Pure EP Uni” shows a large unipolar signal where the entire signal has been captured by the PURE EP™ system (BioSig Technologies, Inc., Minneapolis, MN, USA). The lead labeled “Conv EP Uni” shows a truncated version of the same lead as captured by a conventional EP system with typical gain settings. The truncation is evident from the flat tops and bottoms of the signal. However, when filtering is applied (“Filtered Conv EP Uni” with 50-Hz low-pass filter plus power line notch), as is often done to remove power line and high-frequency noise, the truncation is not evident, and much of the true signal detail is also lost.

**Figure 3:**
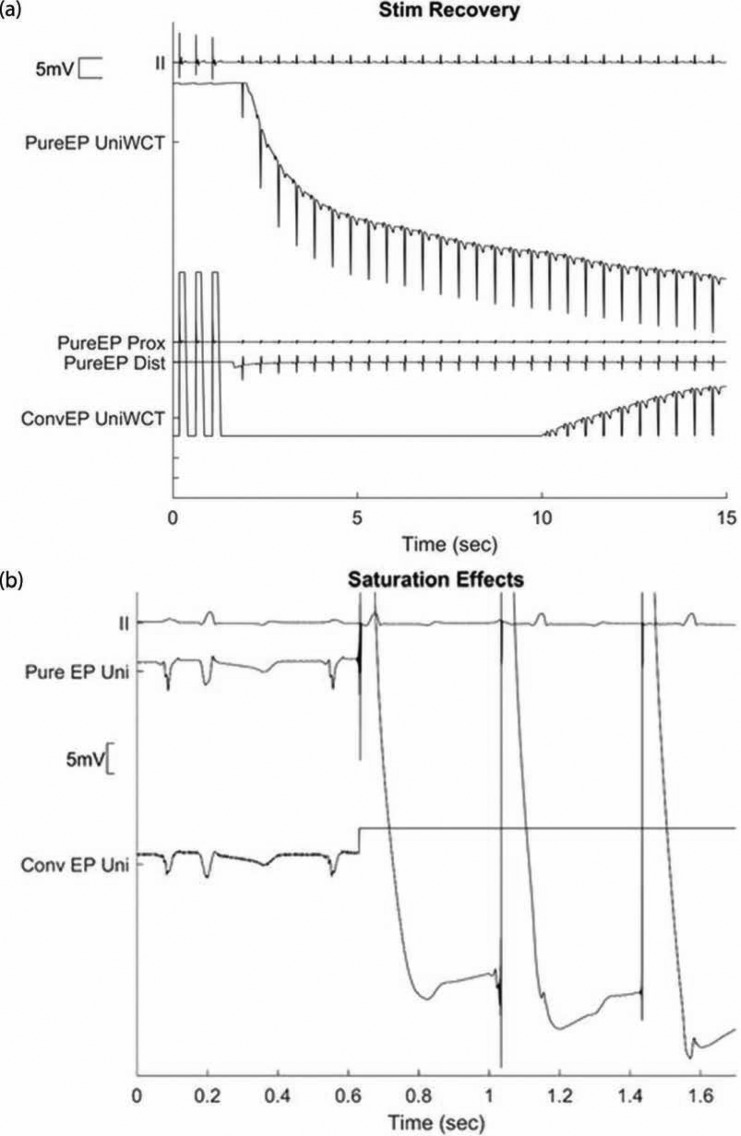
(A) Differences in the times of recovery post-pacing in conventional recording systems versus the PURE EP™ system (BioSig Technologies, Inc., Minneapolis, MN, USA), on application of a pacing stimulus. UniWCT unipolar signal references the Wilson central terminal. Prox: proximal; Dist: distal; ConvEP: conventional EP recorder. (B) Drift in the baseline and saturation upon the application of the unbalanced pacing stimulus to the distal electrode as viewed as a unipolar lead. Pure EP Uni: unipolar signal as recorded by the PURE EP™ system (BioSig Technologies, Inc., Minneapolis, MN, USA); Conv EP Uni: unipolar signal recorded by a conventional recorder that has saturated.

**Figures 3A and 3B** show examples of saturation. Pacing has overdriven both systems into saturation. In the first example, the conventional EP system takes almost 10 s to recover from saturation, while the PURE EP™ system (BioSig Technologies, Inc., Minneapolis, MN, USA), with its larger dynamic range, recovers in about 2 s.

**The clipping solution; or is it the clipping problem?**. It is not uncommon for operators to clip signals to remove overlapping signals on the display screen. This involves clamping the display of signals above a set voltage limit to prevent them from obscuring small signals in adjacent leads. Since this is a display action only, it still allows large signals to be recorded without creating artifacts. This works well, as long as the signals are discrete and separated in time. However, if there are fused signals or signals of equal amplitude present near-simultaneously, there could be a smudged view of the entire complex signal owing to clipping.^18^ This may hide significant features of the signal and may, in extreme cases, change the way the signal is interpreted. These interactions occur randomly, and cannot be anticipated by the operator. One possible solution to this clipping problem is to decrease the gain of the system to minimize saturation effects at the expense of increased system noise. The PURE EP™ system (BioSig Technologies, Inc., Minneapolis, MN, USA) minimizes problems here by virtue of its very low hardware gain, high dynamic range, and good noise performance (1 μV root mean square over the full bandwidth). In the case of software clipping, the high bandwidth ensures visualization, and that any data below the clipping level are not compromised by saturation of the hardware.

**Noise due to ablation.** Ablation involves the application of high-frequency electrical power from an RF ablation generator between the distal electrode of an ablation catheter and a reference patch (dispersive or return electrode) placed on the back of the patient. The electrical resistance to the passage of this current generates heat, which in turn causes localized necrosis by thermocoagulation. Ablation essentially creates an unbalanced energy input into the amplifier connected to the distal electrode of the ablation catheter, resulting in additive noise, baseline shift, and obscuration of the underlying signal data. Additional front-end filters designed to block signals at the ablation frequency are required. Specifically, it is important to use passive filters to reduce ablation noise before it enters the active circuitry to prevent nonlinear effects from propagating through the system.^18–20^

**Figure 4:**
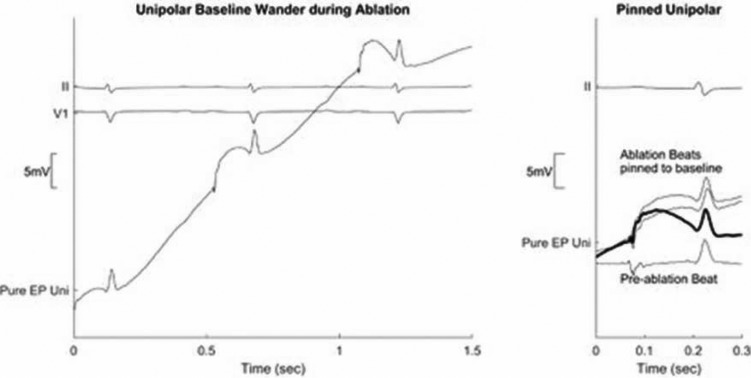
Use of an alternate window to pin the signal of interest to the baseline to help with the evaluation of the changes during the ablation procedure. Pure EP Uni: unipolar signal as recorded by the PURE EP™ system (BioSig Technologies, Inc., Minneapolis, MN, USA); Conv EP Uni: unipolar signal recorded by a conventional recorder, which has saturated.

**Figure 4** shows an example of baseline drift at the onset of ablation. In many cases, the drift can push the signal outside the bounds of the display so that it cannot be viewed. It also shows a solution implemented by the PURE EP™ system (BioSig Technologies, Inc., Minneapolis, MN, USA) for extreme baseline wander via the opening of a new review window that “pins” each beat to the baseline so that the data can be viewed throughout the ablation procedure. The ultimate solution is improved front-end filtering so that acquired and displayed waveforms are visible and clean during the application of RF energy.

**Unipolar signals.** Unipolar signals refer to signals recorded between an electrode placed in the tissue and a neutral reference electrode. In EP recording systems, the preferred reference is the Wilson central terminal (WCT), derived from the electrocardiogram (ECG) limb leads, since this does not require an extra catheter. The unipolar signals are distance-dependent but not direction dependent, making them valuable in mapping and ablating arrhythmias.^17,18,21–23^ Unfortunately, in conventional recording systems, unipolar signals referenced to WCT tend to be noisy. The low cut-off frequency for the highpass filter requires an expanded dynamic range to fully capture the recorded signals with baseline drift. Since the reference is not a dedicated one, the signal is susceptible to noise and surface ECG lead polarization artifact. Extra care thus needs to be taken to ensure the fidelity of the unipolar WCT signal by matching the path of the WCT and the active electrode, which is difficult to achieve in the EP laboratory environment. Other techniques are required to ensure the fidelity of the signal. Filtering of the power line frequency is generally necessary, and filtering of EP laboratory interference may also be required. The use of adaptive notch filters that do not add artifact to this unipolar signal offers the best solution to maintaining the fidelity and usefulness of the unipolar WCT signal.

**Figure 5:**
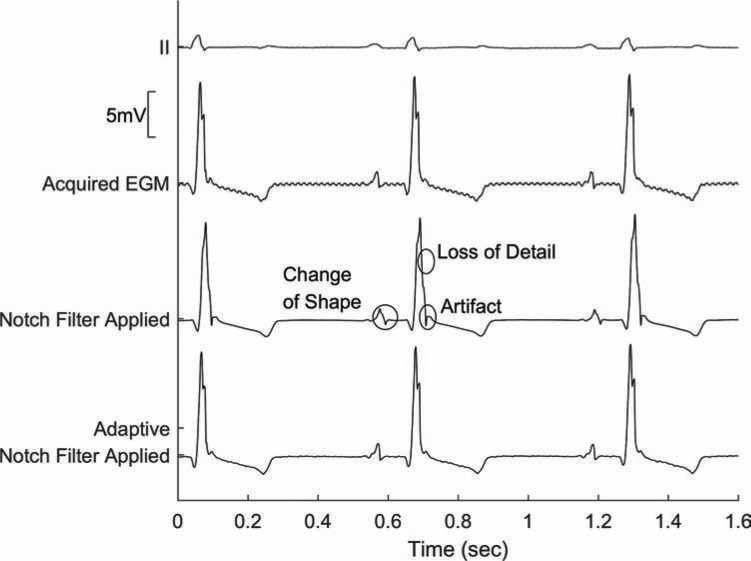
Change in the signal morphology with the application of a classic notch filter (Notch Filter Applied) versus the application of the adaptive notch (Adaptive Notch Filter Applied) compared with the baseline signal morphology (Acquired EGM).

**Figure 5** shows an example of a unipolar signal with fixed frequency noise labeled “Acquired EGM.” This noise can be removed by a conventional notch filter, but this leads to a loss of detail, the development of artifacts, and changes in the shape of the waveform features, as shown in the lead labeled “Notch Filter Applied.” The PURE EP™ system (BioSig Technologies, Inc., Minneapolis, MN, USA) uses a different approach with proprietary adaptive notch filtering that removes the noise without affecting the base waveform shape, as shown in the lower lead of the figure. This preserves the integrity of the WCT referenced lead.

**Contact force.** Maintaininggood contact with tissue remains one of the greatest challenges in modern electrophysiology. It influences mapping and ablation and remains one of the most important factors in determining the success or failure of a procedure. Rightly so, there has been acceleration in innovation to assess contact with tissue.^24,25^ Current technology requires the use of relatively stiff catheters to transmit the force to the force sensor effectively. In addition, the angle of contact made by the distal electrode with the cardiac tissue and the effect of the intracardiac structures coming into contact with the catheter system may produce significant variability.^26^ The sampling rate of the contact force data and the stability of contact remain as parameters that affect the contact force readings.

**Figure 6:**
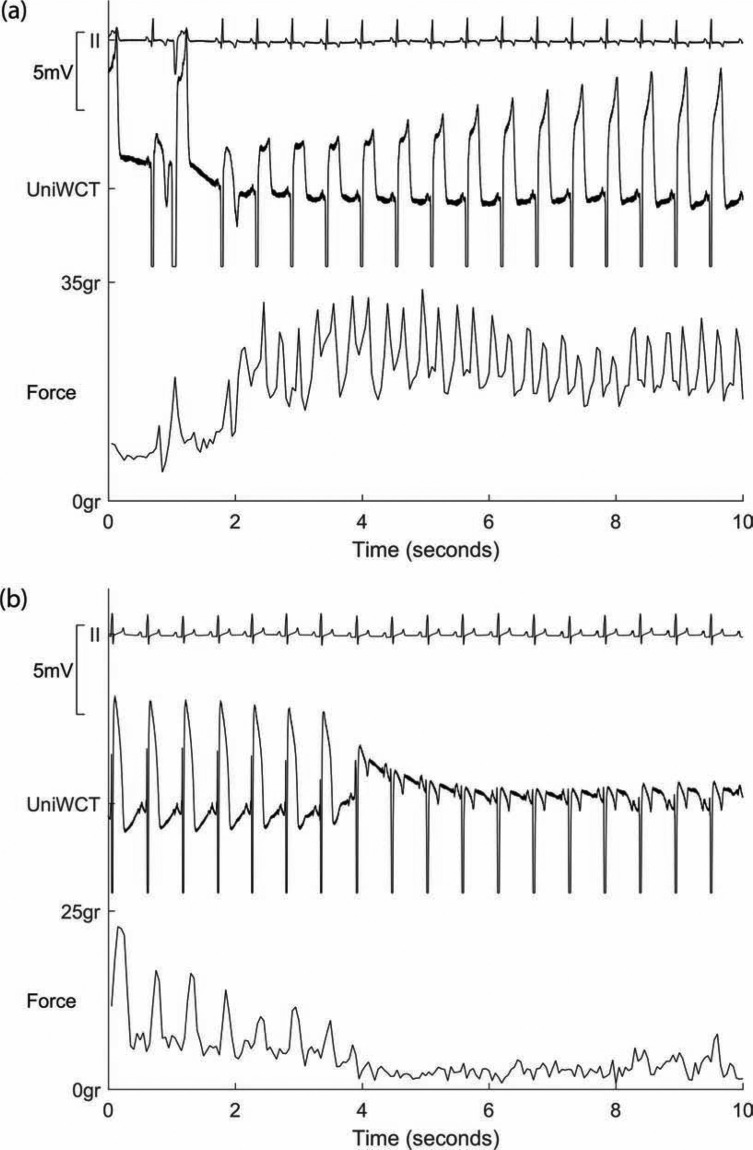
(A,B) Graded increase in the current of injury on the unipolar electrogram, as there is an increase in the force of contact and near-simultaneous decrease in it, with an accompanying decrease in the contact force. UniWCT: unipolar signal referencing the Wilson central terminal; Force: force as estimated by the ThermoCool^®^ SmartTouch^®^ catheter (Biosense Webster, Diamond Bar, CA, USA).

However, with the expanded frequency and dynamic range of the PURE EP™ system (BioSig Technologies, Inc., Minneapolis, MN, USA), the current of injury (COI) caused by the catheter contact with tissue provides a good estimate of contact force without the need for stiff catheters. This reflects the local injury to the tissue caused by contact of the catheter. The degree of local injury is proportional to the amount of contact force applied. This COI is reversible, and correlates well with standard contact force-sensing catheters **(Figures 6A and 6B)**.

**Poor catheter visualization.** Withthe increase in the ablation of arrhythmias from the sinuses of Valsalve, there are significant concerns about injuring the conduction system and the coronary arteries at this location. It is imperative to be able to distinguish catheter position above and below the aortic valve in order to avoid injury to the coronaries. Currently, no reproducible way to do that exists.

Regions at the base of the heart remain largely dependent on accurate positioning of an intracardiac echocardiography probe for their visualization.^27^ However, respiratory and cardiac motion, coupled with catheter artifact, contribute to poor images in this location.^16^ Imaging is also relatively insensitive to small changes in the position of the catheter tip during ablation. It is extremely important to be able to visualize the catheter tip in these locations, owing to the clustering of important structures in the vicinity.

**Figure 7:**
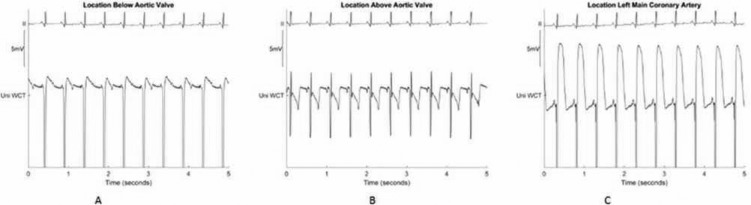
Changes in the unipolar electrogram (EGM) characteristics depending on the position of the catheter tip. UniWCT: unipolar signal referencing the Wilson central terminal. (A) The current of injury is positive when in contact with the tissue below the aortic valve. However, above the aortic valve, within the sinuses of Valsalva, the current of injury is different despite similar contact (B). (C) The ventricularization of the unipolar EGMs compared with those in positions in (A) and (B) corresponds to a location within the coronary artery.

Using the PURE EP™ system (BioSig Technologies, Inc., Minneapolis, MN, USA), distinctive changes in unipolar signal EGM morphology were noted when ablating above the valve compared with ablating below the valve or within the coronary artery **(Figure 7)**. While recording these signals, we confirmed the catheter position and orientation using intracardiac echocardiography and fluoroscopy. We also used contrast to confirm the position of the catheter within the coronary artery. We believe that the unipolar recordings made were suggestive of COI, and given appropriate filtering, frame rate, and sampling rate, they could be reproduced in any system.

The PURE EP™ system (BioSig Technologies, Inc., Minneapolis, MN, USA) allows for the display of signals with more than one filter at the same time, a feature not found in current systems. This allows for the ability to look at signals filtered in multiple ways for specific reasons. Further, the finding that we may be able to make a distinction between ventricularized signals within the coronary artery vs. contact EGMs above and below the valve is novel. This is being further evaluated for consistency. The ability to predict the location of the catheter can be used as an extra checkpoint along with intracardiac echocardiography and fluoroscopy prior to ablation.

Our hypothesis for the change in the EGMs is that, given that COI emanates from myocardial injury with contact, no COI changes were noted above the valve owing to the dominant muscle mass being present below the valve directing the resultant COI away from the point of contact. This causes a negative COI at this location. In contrast, below the valve and from within the coronary arteries, the COI originates from the local contact within or on the myocardium, giving rise to a typical positive COI from that location.

**Conduction tissue signal recording.** Conduction tissue within the distal ventricles remains intimately associated with the muscle. Mapping these signals with a bipolar electrode is difficult because there is fusion and overlapping of signals originating from the muscle and the conduction system. Arrhythmias originating from the conduction system, therefore, are more difficult to map, since successful annotation of the smaller signal within the bigger myocardial signal needs considerable experience, and may not always be possible. The assessment of ablation lesions targeting any of these two components remains problematic.

**Figure 8:**
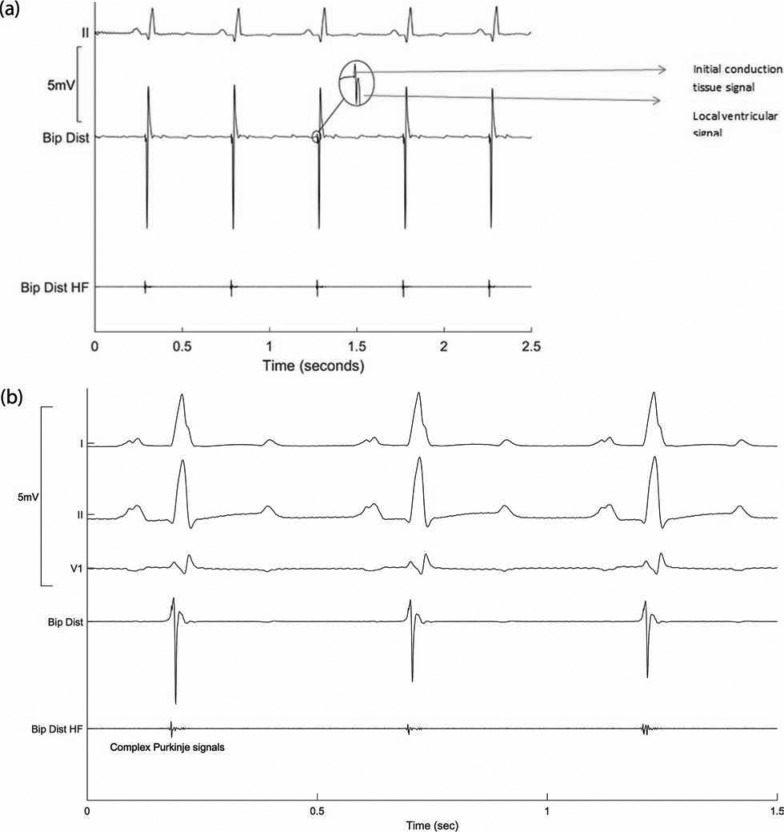
(A) Use of a high-pass filter to selectively show signals pertaining to the conduction system separate from the local myocardial signal. The inset shows the composite signal consisting of the initial component of conduction tissue and the local ventricular signal. (B) Display of signals filtered in multiple ways for specific reasons during mapping. The signal recorded from the tip of the papillary muscle shows complex signals corresponding to conduction system activity. Bip Dist: bipolar distal; Bip Distal HF: bipolar distal high-frequency filter applied. Bip Dist is processed with a standard 30-Hz high-pass signal filter, and Bip Dist HF is processed with a 200-Hz high-pass filter, plus proprietary processing to suppress unwanted transients.

A distinguishing feature of signals from the conduction system is their high-frequency content. This difference allows for these signals to be selectively displayed by using a high-pass filter. In the PURE EP™ system (BioSig Technologies, Inc., Minneapolis, MN, USA), this can be done as a separately displayed item to show the presence of signals from the conduction system. However, one of the problems in using a simple linear filter is the transient response to impulse-like signals produced by the myocardium.^28^ Large local sharp spikes produced during depolarization will cause the filter to “ring” at its resonant frequency and produce false positives. Additional processing is required to suppress these unwanted transient responses **(Figures 8A and 8B)**.

## Future directions

1. *Reliable marker for transmurality of lesion.* We do not have any reliable measures of lesion transmurality. The use of surrogate measures like drop in impedance, changes in the COI, and change in EGM need to be integrated into a definitive variable for its determination. In addition, the effects of ablation upon the local unfiltered unipolar EGM, to help discern the effects of ablation in creating a transmural lesion, remains to be evaluated.
2. *EGM-based contact force estimation.* There has been a correlation noted between the amount of contact force and the rise in the COI. However, changes in the COI have not been standardized for radius of the catheter tip, the difference between the COI in the atrium and ventricle, the effect of intracavitary structures on the COI, or the standardization of COI at the annular region.
3. *Discerning the underlying substrate based upon the recorded EGM.* Currently, there are only approximations made to evaluate the underlying substrate when mapping cardiac tissue. There are no EGM characteristics that are able to recognize and differentiate between scar tissue, fat, or myocardium consistently in all circumstances. Using the better fidelity in bipolar and unipolar characteristics of the PURE EP™ system (BioSig Technologies, Inc., Minneapolis, MN, USA) we hope to be able to create areas of scar and map them to identify unique characteristics of local EGMs.
4. *Evaluate the performance of current automated mapping algorithms.* The PURE EP™ system (BioSig Technologies, Inc., Minneapolis, MN, USA) can reproduce recorded cardiac signals with greater fidelity than existing commercial systems. Current algorithms are not very efficient in discerning between components of an EGM, especially in multicomponent recordings. With the PURE EP™ system (BioSig Technologies, Inc., Minneapolis, MN, USA), we plan to reevaluate the current automated algorithms for accuracy in the annotation of EGMs, and perhaps clarify the complex process of mapping and tagging points correctly.
5. *Application of the system in other areas of signal recording and processing.* Signal recording and processing is an integral part of various mapping techniques in fields other than cardiology. We hope to apply the expanded hardware configurations of the system for mapping and recording signals in fields like neurology and ophthalmology, in order to better be able to characterize regional electrical activity.

## Acknowledgment

We thank Jennifer Mears for her help in preparing and proofreading this manuscript.
